# Public reaction to Chikungunya outbreaks in Italy—Insights from an extensive novel data streams-based structural equation modeling analysis

**DOI:** 10.1371/journal.pone.0197337

**Published:** 2018-05-24

**Authors:** Naim Mahroum, Mohammad Adawi, Kassem Sharif, Roy Waknin, Hussein Mahagna, Bishara Bisharat, Mahmud Mahamid, Arsalan Abu-Much, Howard Amital, Nicola Luigi Bragazzi, Abdulla Watad

**Affiliations:** 1 Department of Medicine 'B', Sheba Medical Center, Tel-Hashomer, Israel; 2 Sackler Faculty of Medicine, Tel-Aviv University, Tel-Aviv, Israel; 3 Azrieli Faculty of Medicine, Bar-Ilan University, Zefat, Israel; 4 Padeh and Ziv Medical Centers, Zefat, Israel; 5 The Zabludowicz Center for Autoimmune Diseases, Sheba Medical Center, Tel-Hashomer, Israel; 6 Society for Health Promotion of the Arab Community, Nazareth, Israel; 7 Endoscopy Unit of the Nazareth Hospital EMMS, Nazareth, Israel; 8 School of Public Health, Department of Health Sciences, University of Genoa, Genoa, Italy; University of California Davis, UNITED STATES

## Abstract

The recent outbreak of Chikungunya virus in Italy represents a serious public health concern, which is attracting media coverage and generating public interest in terms of Internet searches and social media interactions. Here, we sought to assess the Chikungunya-related digital behavior and the interplay between epidemiological figures and novel data streams traffic.

Reaction to the recent outbreak was analyzed in terms of Google Trends, Google News and Twitter traffic, Wikipedia visits and edits, and PubMed articles, exploiting structural modelling equations.

A total of 233,678 page-views and 150 edits on the Italian Wikipedia page, 3,702 tweets, 149 scholarly articles, and 3,073 news articles were retrieved. The relationship between overall Chikungunya cases, as well as autochthonous cases, and tweets production was found to be fully mediated by Chikungunya-related web searches. However, in the allochthonous/imported cases model, tweet production was not found to be significantly mediated by epidemiological figures, with web searches still significantly mediating tweet production. Inconsistent relationships were detected in mediation models involving Wikipedia usage as a mediator variable. Similarly, the effect between news consumption and tweets production was suppressed by the Wikipedia usage. A further inconsistent mediation was found in the case of the effect between Wikipedia usage and tweets production, with web searches as a mediator variable. When adjusting for the Internet penetration index, similar findings could be obtained, with the important exception that in the adjusted model the relationship between GN and Twitter was found to be partially mediated by Wikipedia usage. Furthermore, the link between Wikipedia usage and PubMed/MEDLINE was fully mediated by GN, differently from what was found in the unadjusted model.

**In conclusion**—a significant public reaction to the current Chikungunya outbreak was documented. Health authorities should be aware of this, recognizing the role of new technologies for collecting public concerns and replying to them, disseminating awareness and avoid misleading information.

## Introduction

Chikungunya virus is a small, enveloped, positive-sense, single-stranded RNA alphavirus, a member of the *Togaviridae* family [1]. Chikungunya virus can cause an acute febrile disease associated with skin rash and severe arthralgia [2]. The virus is generally transmitted to humans from the bite of infected female mosquitoes (*Aedes aegypti* and *Aedes albopictus*).

During the last decades, several outbreaks of Chikungunya infection were described worldwide, including the Americas, islands in the Indian Ocean, and Europe, secondary to change of distribution and habitat of *Aedes* mosquitoes [3–7].

Since the emergence of the first cases of Chikungunya in Italy in 2007, various outbreaks in Europe were described [4, 6]. Recently, an outbreak of Chikungunya has been reported in the central Italy since August 2017 [8]. The first three confirmed cases of the recent outbreak occurred in the province of Rome, in the Lazio region. By September 20, 2017, eighty-six confirmed autochthonous Chikungunya cases were detected by the regional surveillance system. The reemergence of Chikungunya in Italy in the last decade is considered to be mediated by *Aedes* mosquitoes, a vector that is widely dispersed in Italy.

The internet represents a major source of health-related information that is highly accessible by all users. Stemming from the fact that most published content is unregulated, the potential for divulging and spreading false information, for instance unexpected outbreaks and epidemics, remains unmet with content regulation [9, 10].

The importance of Internet content and search as a tool for the surveillance of outbreaks has been previously reported. Brownstein et al. [11] showed that during the peanut butter-associated outbreak of *Salmonella enterica* subtype *Typhimurium*, search activity measured by Google Trends (GT) provided preliminary evidence of an emerging problem enabling early disease detection. Similar findings were reported in several studies regarding the epidemics of the flu studying web search trends [12–14].

Following the H1N1 pandemic of 2009, Chew and Eysenbach [15] archived and analyzed 2 million messages on Twitter (“tweets”) related to the pandemic, concluding that they can be used for real-time content analysis, which could potentially allow health authorities to deal with public concerns. The same conclusion was demonstrated during the Ebola outbreak by other authors [16, 17].

Novel data streams, such as social networks or website searches, provide a solid platform for tracking people's behaviors in real time concerning health-related issues. Results devised from search data provide integral tools for healthcare scientists interested in analyzing behaviors towards medical conditions. Moreover, novel data streams allow for the assessment of public interest, concerns, engagement, and perception, which would otherwise remain unmonitored by classical surveillance approaches.

Data processing and analysis have been extensively implicated in medical research. However, dealing with data generated from novel data streams is challenging because of their technically innovative features [18–21].

The aim of this study was to assess the digital behaviors and complex interplay between novel data streams induced by the recent Chikungunya outbreak in Italy.

## Materials and methods

We analyzed Internet data through several novel data streams, notably from GT, Wikipedia, Twitter, PubMed/MEDLINE, and Google News (GN). All novel data streams utilized and units of measurement/ranges of values in the current study are briefly overviewed in Table 1. All data are unbounded count variables, with the exception of data generated by GT and GN, which are provided as rescaled in the range 0–100.

**Table 1:** Novel data streams utilized in the current study.

Novel data streams	Study period	Details	Range
Wikipedia	2004–2017	Number of edits performed	Unbounded
Wikipedia	2015–2017	Number of pageviews	Unbounded
Twitter	2006–2017	Number of tweets produced	Unbounded
Google Trends	2004–2017	Search volumes carried out	0–100
PubMed/MEDLINE	2004–2017	Number of articles written	Unbounded
Google News	2008–2017	Volumes of news consumed	0–100

GT is a free online open-source tracking system of Internet search activity. In the current investigation, GT has been used to assess public interest in Chikungunya-related issues. For this purpose, GT was mined from inception (last search carried out on October 19, 2017). Searches on GT can be performed using the “search term” or the “search topic” options. The first approach enables to search exactly the keyword(s) entered by the user, while the second option results in a broader search where GT systematically performs a search of all web searches containing the entered keyword(s) or related pertinent terms.

GT web queries are reported not as absolute, raw figures but as normalized figures (relative search volumes or RSVs). In detail, in order to make comparisons, every query is divided by the total searches performed in that given region and time range, then re-scaled on a scale from 0 to 100 based on the topic’s proportion with respect to all searches carried out on all searchable topics.

In our analysis, we used the second searching option. In particular, we looked for “Chikungunya (Topic)” and limited the search within Italy. For further details concerning GT, the reader is referred to Nuti et al’s review of GT [22].

Wikipedia is a free online encyclopedia launched in 2001. It is generally one of the most visited websites worldwide and often consulted for health-related information. We looked at the number and time of edits for the Italian Wikipedia entry for “Chikungunya” between 2004 and 2017, as well as page visits between July 2015 and October 2017 using the Wikipedia page’s revision history and the Wikimedia Foundation’s Pageviews Analysis tools [23], respectively. The chronological changes of the Wikipedia page were assessed on October 19, 2017.

Twitter is a social media and news platform where users post and interact with messages “tweets”. A Twitter search for “Chikungunya” in Italy was performed to compare the number and time of tweets with Chikungunya outbreaks between 2006 and 2017. The search was performed and results were identified manually on October 19, 2017 and classified by number of tweets per year.

PubMed is an online repository of scholarly peer-reviewed articles using MEDLINE, a large bibliographic database covering almost all medical fields and disciplines. A PubMed/MEDLINE search was performed on October 19, 2017 for all Chikungunya-related peer-reviewed articles written in Italy and/or by at least one Italian scholar as co-author.

GN is a free news aggregator provided and operated by Google, selecting articles from thousands of news websites. It was first launched in 2002 as beta version and released officially in 2006. GN regarding Chikungunya-related issues in Italian language between 2008 and 2017 were searched and identified manually on October 19, 2017 and classified by number of news per year.

Correlational analyses and multivariate regression models were performed on all the novel data streams described above with the number of Chikungunya infection cases.

The partial least squares path modeling (PLS-PM) method to structural equation modeling (SEM) was chosen, in that it allows estimating complex cause-effect relationship models with latent variables, being a component-based estimation approach. ‬

According to MacKinnon and collaborators [24], a suppressor effect can be found in the case of “a variable which increases the predictive validity of another variable (or set of variables) by its inclusion in a regression equation”. Rucker and colleagues [25] have defined a suppressor variable “as one that undermines the total effect by its omission, meaning accounting for it in a regression equation enhances the predictive utility of the other variables in the equation”. We used this statistical model to estimate cause-effect relationships between the different online sources used and the number of confirmed autochthonous cases, notified autochthonous cases, and allochthonous/imported cases of Chikungunya infection during the recent 2017 outbreak.

PLS-PM models have been conducted both unadjusting and adjusting for the Internet penetration index, in order to avoid that the increase of searches on the topic may be in part due to the increase of users instead of an increasing interest on the topic. Since this fact could directly affect the results obtained (in the study period, the number of Internet users has exponentially grown in recent years, growing from 33.2% to 66.0%), both models are hereby presented. Data related to the Internet penetration index were taken from the National Institute for Statistics (ISTAT).

All statistical analyses were carried out using the commercial software XLSTAT Premium (version 19.7, Addinsoft, France).

All figures with p-value less than 0.05 were considered statistically significant.

## Results

A *corpus* of 233,678 pageviews and 150 edits on the Italian Wikipedia page, 3,702 tweets written in Italian, 149 scholarly peer-reviewed articles from Italy or by Italian scholars, and 3,073 news articles written in Italy and/or in Italian language were found and analyzed.

**Fig 1:**
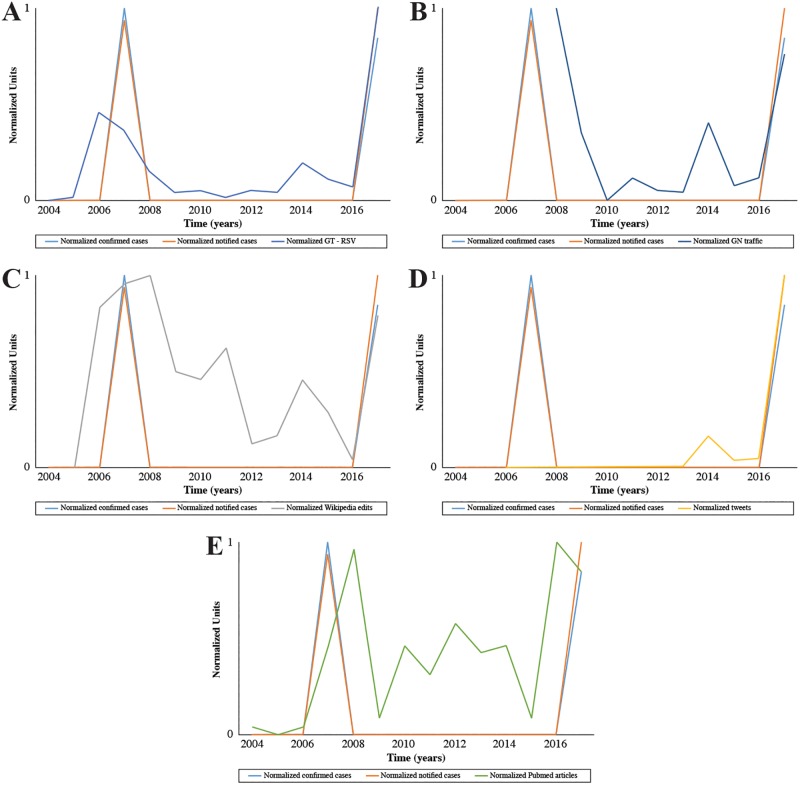
Time trend of Chikungunya-related web search volumes as captured by Google Trends (1A), Google News traffic (1B), Wikipedia edits (1C), tweets (1D), scholarly peer-reviewed articles indexed in PubMed (1E) and correlation with epidemiological cases (notified and confirmed).

The correlational analyses between the different novel data streams used and the number of notified or confirmed cases of Chikungunya infection showed several significant temporal correlations (Table 2); notably, a significant correlation was observed between the RSVs on GT and notified cases (p = 0.0008), as well as with confirmed cases (p = 0.0030). There was an initial burst of web searches for Chikungunya in 2006 and 2007, a second smaller one in 2014, and a very large peak in 2017 (Fig 1A). There was also a significant correlation between notified cases and tweets (p = 0.0171). With 3,702 Chikungunya-related tweets shared in the past 12 years, Twitter activity showed a small spike in tweets in 2014 and a very large one in 2017 (Fig 1D).

**Table 2:** Correlation between novel data streams traffic and epidemiological cases of Chikungunya virus infection (notified and confirmed).

Novel data streams	Notified cases		Confirmed cases	
	Correlation coefficient	Statistical significance	Correlation coefficient	Statistical significance
Wikipedia edits	0.51	0.0624	0.52	0.0566
Twitter	0.67	0.0171*	0.57	0.0530
Google Trends	0.79	0.0008***	0.73	0.0030**
PubMed/MEDLINE	0.28	0.3323	0.31	0.2808
Google News	0.48	0.1603	0.48	0.1603

*statistically significant with p-value less than 0.05;

**statistically significant with p-value less than 0.01;

***statistically significant with p-value less than 0.001.

While the other sources of online data did not show a significant correlation with the number of cases, we observed similar spikes in online activity as was seen with GT and Twitter. Over 3,000 news articles were written and aggregated on GN since 2008. Small bursts in traffic were observed in 2011 and 2014, and large ones in 2008 and 2017 (Fig 1B).

The Italian “Chikungunya” Wikipedia page was created in 2006 and underwent through 150 modifications by users and has been viewed 244,358 times. It underwent most edits during its year of creation and the two subsequent years. The page gradually saw less modifications except for small bursts of edits in 2011 and 2014. However, in 2017 the number of edits spiked back up to ranges close to that of the page’s inception (Fig 1C). Between July 2015 and August 2017, there was a daily average of 69 pageviews. However, between September and October 2017 there was a very large burst of page traffic, resulting in an average of 3,862 daily active visits.

A PubMed/MEDLINE search of academic works written in Italy and/or in Italian yielded 149 peer-reviewed articles between 2004 and 2017. Major spikes in number of publications could be seen in 2008 and 2017, with a more than average amount being written between 2010 and 2014 (Fig 1E).

Correlations between novel data streams and allochthonous/imported cases of Chikingunya were not statistically significant (data not shown).

Concerning the PLS-SEM approach, global R^2^ was 0.469 and 0.663 for the notified cases (unadjusted and adjusted models, respectively), 0.453 and 0.658 for the confirmed cases (unadjusted and adjusted models, respectively), 0.371 and 0.666 for the imported cases (unadjusted and adjusted models, respectively), indicating a satisfactory fitting of the computed models. Notably, the fitting parameter (global R^2^) was higher for the adjusted models which takes into account the Internet penetration index. Further details are reported in Table 3.

**Table 3:** Structural equation modelling for confirmed, notified and imported cases of Chikungunya virus.

Latent variable	Confirmed cases (unadjusted model)		Confirmed cases (adjusted model)		Notified cases (unadjusted model)		Notified cases (adjusted model)		Imported cases (unadjusted model)		Imported cases (adjusted model)	
	R^2^	Adjusted R^2^	R^2^	Adjusted R^2^	R^2^	Adjusted R^2^	R^2^	Adjusted R^2^	R^2^	Adjusted R^2^	R^2^	Adjusted R^2^
PubMed/MEDLINE	0.077	0.077	0.077	0.077	0.094	0.094	0.094	0.094	0.036	0.036	0.036	0.036
GN	0.206	0.139	0.417	0.369	0.221	0.156	0.419	0.370	0.186	0.118	0.370	0.317
Wikipedia edits	0.472	0.375	0.913	0.898	0.451	0.351	0.909	0.893	0.353	0.236	0.991	0.989
GT	0.636	0.527	0.919	0.895	0.700	0.610	0.928	0.907	0.405	0.227	0.946	0.930
Twitter	0.874	0.818	0.965	0.949	0.878	0.823	0.963	0.946	0.875	0.820	0.987	0.981
Mean	0.453		0.658		0.469		0.663		0.371		0.666	

**Fig 2:**
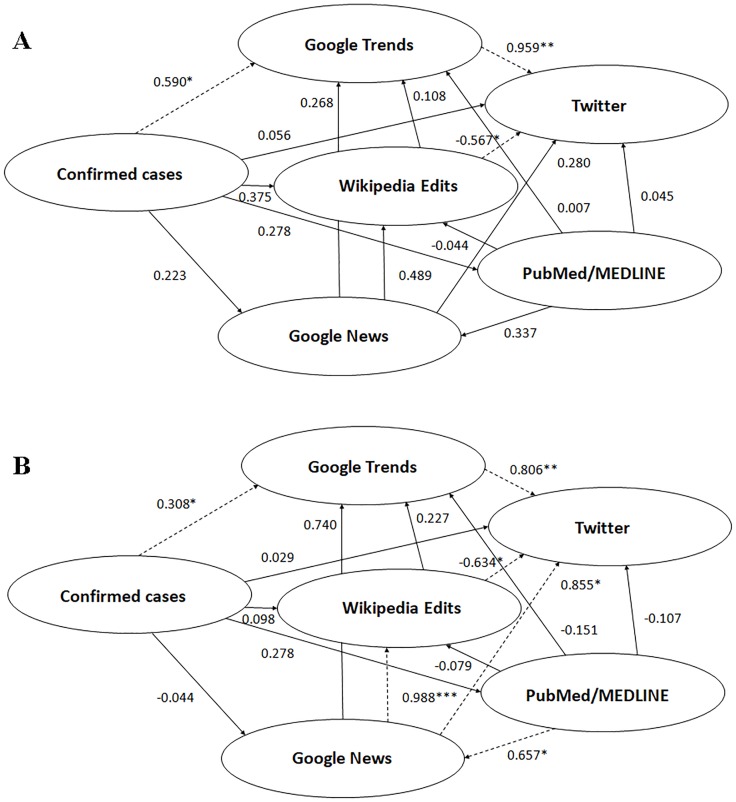
Structural equation modeling for confirmed autochthonous cases of Chikungunya showing the relationships among the novel data streams used in the current study, unadjusted model (2A) and adjusted model (2B) for the internet penetration index. *statistically significant with p-value less than 0.05; **statistically significant with p-value less than 0.01; ***statistically significant with p-value less than 0.001.

**Fig 3:**
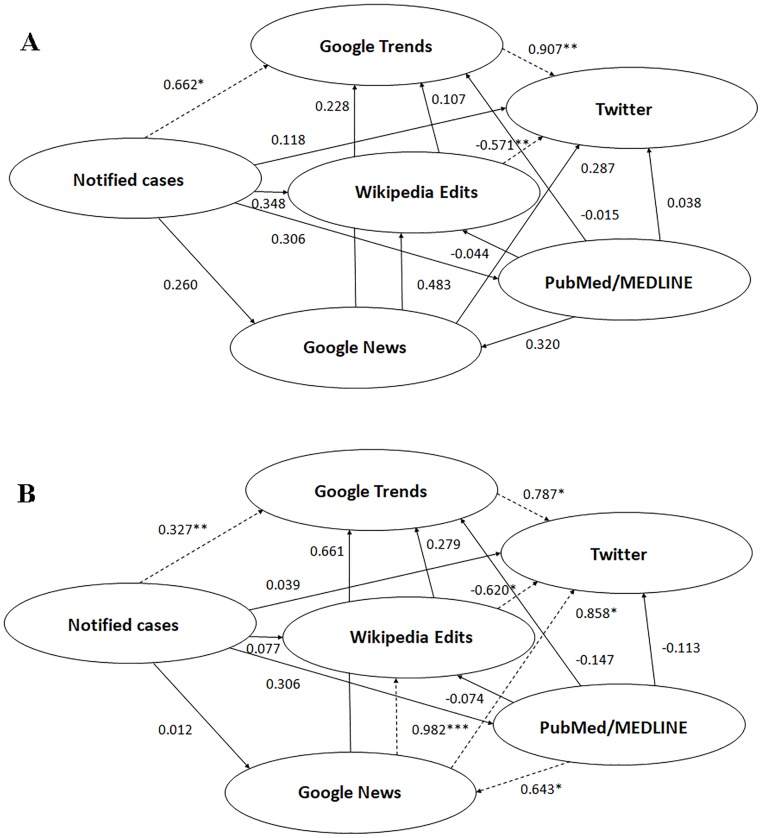
Structural equation modeling for notified autochthonous cases of Chikungunya showing the relationships among the novel data streams used in the current study, unadjusted model (3A) and adjusted model (3B) for the internet penetration index. *statistically significant with p-value less than 0.05; **statistically significant with p-value less than 0.01; ***statistically significant with p-value less than 0.001.

**Fig 4:**
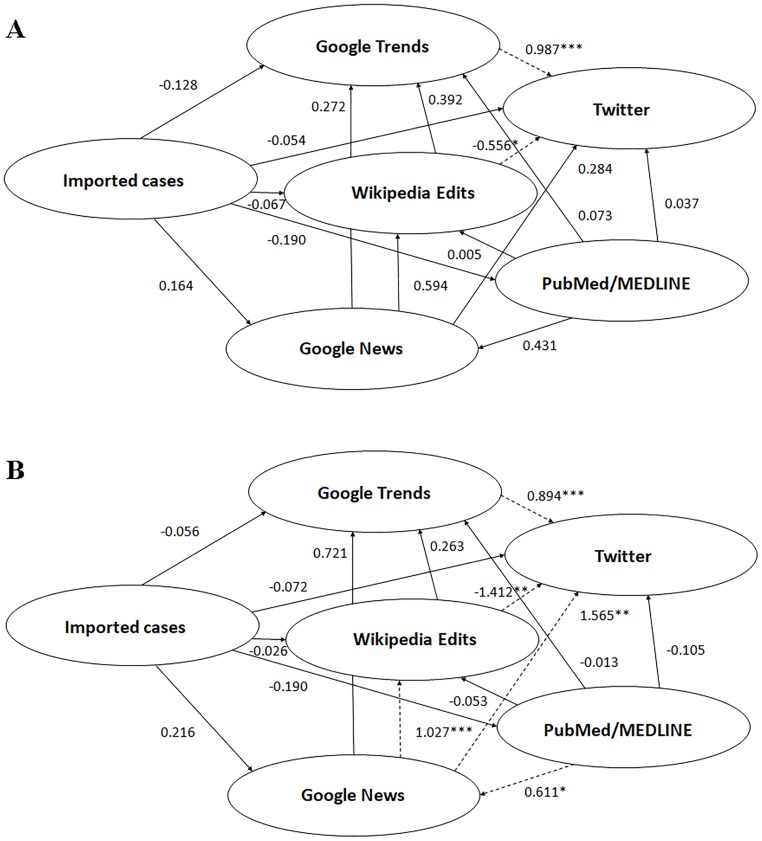
Structural equation modeling for allochthonous/imported cases of Chikungunya showing the relationships among the novel data streams used in the current study, unadjusted model (4A) and adjusted model (4B) for the internet penetration index. *statistically significant with p-value less than 0.05; **statistically significant with p-value less than 0.01; ***statistically significant with p-value less than 0.001.

Regarding the unadjusted model of the PLS-SEM approach, the relationship between epidemiological autochthonous Chikungunya cases, either notified or confirmed, and tweets production was found to be fully mediated by the Chikungunya-related web searches as captured by GT (path coefficient between autochthonous confirmed cases and GT 0.590, p<0.05, and between GT and Twitter 0.959, p<0.01—Fig 2A; path coefficient between autochthonous notified cases and GT 0.662, p<0.05, and between GT and Twitter 0.907, p<0.01—Fig 3A). However, in the allochthonous/imported cases model, tweet production was not found to be significantly mediated by the epidemiological cases, but web searches as described by GT still significantly mediated tweet production (path coefficient between imported cases and GT -0.128, p>0.05, and between GT and Twitter 0.987, p<0.01—Fig 4A).

Taking into account the Internet penetration index (adjusted model), the relationship between epidemiological autochthonous Chikungunya cases, either notified or confirmed, and tweets production remained fully mediated by the Chikungunya-related web searches captured by GT (path coefficient between autochthonous confirmed cases and GT 0.308, p<0.05, and between GT and Twitter 0.806, p<0.01—Fig 2B; path coefficient between autochthonous notified cases and GT 0.327, p<0.01, and between GT and Twitter 0.787, p<0.05—Fig 3B). Also in the allochthonous/imported cases model, adjusted for the internet penetration index, tweet production was still found not to be significantly mediated by the epidemiological cases, but web searches described by GT still significantly mediated tweet production (path coefficient between imported cases and GT -0.056, p>0.05, and between GT and Twitter 0.894, p<0.001—Fig 4B).

In the unadjusted model, inconsistent relationships were detected in mediation models involving Wikipedia usage as a mediator variable. The direct effect between epidemiological cases and tweets production was found to be suppressed by the editing of Wikipedia (path coefficient between epidemiological confirmed cases and Wikipedia 0.375, p>0.05, and between Wikipedia and Twitter -0.567, p<0.05—Fig 2A; path coefficient between epidemiological notified cases and Wikipedia 0.348, p>0.05, and between Wikipedia and Twitter -0.571, p<0.01—Fig 3A; path coefficient between allochthonous/imported cases and Wikipedia -0.067, p>0.05, and between Wikipedia and Twitter -0.556, p<0.05—Fig 4A). A proof of such suppressor effect was obtained by calculating the regression coefficient of epidemiological cases as a predictor of tweets production (regression coefficients *c* 0.108 and 0.053 for the notified and confirmed cases models, respectively, being smaller in both cases than the computed path coefficients *c’* 0.118 and 0.056).

These findings related to the suppressor effect of Wikipedia held in the adjusted models (path coefficient between epidemiological confirmed cases and Wikipedia 0.098, p>0.05, and between Wikipedia and Twitter -0.634, p<0.05—Fig 2B; path coefficient between epidemiological notified cases and Wikipedia 0.077, p>0.05, and between Wikipedia and Twitter -0.620, p<0.05—Fig 3B; path coefficient between allochthonous/imported cases and Wikipedia -0.026, p>0.05, and between Wikipedia and Twitter -1.412, p<0.01—Fig 4B).

Similarly, in the unadjusted model, the direct effect between scientific interest (assessed using bibliometric index as a proxy) and tweets production was suppressed by Wikipedia usage (path coefficient between PubMed/MEDLINE -0.044, p>0.05 both for the notified and confirmed cases models, Figs 2A and 3A). The regression coefficient *c* was 0.004, smaller than the two path coefficients *c’* 0.038 and 0.045, for the notified and confirmed cases models, thus confirming the suppressor effect. These findings remained valid when incorporating the Internet penetration index in the model (adjusted model): the path coefficient between PubMed/MEDLINE and Wikipedia yielded a value of -0.079 and -0.074, for confirmed and notified cases of Chikungunya, respectively (Figs 2B and 3B). A similar trend was obtained for allochthonous/imported cases (path coefficient between PubMed/MEDLINE and Wikipedia 0.005, p>0.05, and -0.053, p>0.05, for unadjusted and adjusted models, respectively, as shown in Fig 4A and 4B).

Similarly, in the unadjusted model, the direct effect between news consumption (as assessed by GN) and tweets production was suppressed by the Wikipedia usage (path coefficients between GN and Wikipedia 0.483, p>0.05, for the notified cases model; 0.489, p>0.05, for the confirmed cases model; Figs 2A and 3A). Once more, the regression coefficient *c* 0.046 was smaller than the path coefficients *c’* 0.287 and 0.280 in the notified and confirmed cases models, respectively. However, this finding could not be replicated in the adjusted model: the path coefficient between GN and Wikipedia yielded a value of 0.982, p<0.001, and 0.988, p<0.001, for the notified and confirmed cases models, respectively (Figs 2B and 3B). A similar discrepancy between the unadjusted and the adjusted model could be detected for allochthonous/imported cases of Chikungunya: path coefficient between GN and Wikipedia 0.594, p>0.05, and 1.027, p<0.001, respectively (Fig 4A and 4B).

In the unadjusted model, a further inconsistent mediation was found in the case of the effect between Wikipedia usage and tweets production, with web searches (as captured by GT) as a mediator variable. The path coefficients between Wikipedia and GT and between GT and Twitter were 0.107, p>0.05, and 0.907, p<0.01, respectively for the notified cases model, and 0.108, p>0.05, and 0.959, p<0.01, for the confirmed cases model (Figs 2A and 3A). The effect of Wikipedia usage on tweets production was, as already said, significantly negative. Similar findings were reported in the adjusted model: the path coefficients between Wikipedia and GT and between GT and Twitter yielded a value of 0.227, p>0.05, and 0.806, p<0.01, for the confirmed cases model, and 0.279, p>0.05, and 0.787, p<0.05, for the notified cases model. A similar statistical pattern could be found for the allochthonous/imported cases of Chikungya: the path coefficients were computed 0.392, p>0.05, and 0.987, p<0.001, and 0.263, p>0.05, and 0.894, p<0.001, for the unadjusted and adjusted models, respectively (Fig 4A and 4B).

All path coefficients with their standard errors, T-statistics, p-value, the computed bootstrapped values and standard errors, critical ratio, lower and upper bound values are reported in Tables 4 to 9 (even-numbered tables for unadjusted and odd-numbered for adjusted models).

**Table 4:** Structural equation modeling for confirmed cases of Chikungunya virus (unadjusted model).

Latent variable	Value	Standard error	T	Pr > |t|	f^2^	Value Bootstrap	Standard error Bootstrap	Critical ratio (CR)	Lower bound (95%)	Upper bound (95%)
**Twitter**										
Confirmed cases	0.056	0.193	0.292	0.778	0.011	-0.084	0.533	0.106	-1.660	1.048
PubMed/MEDLINE	0.045	0.139	0.324	0.754	0.013	-0.064	0.309	0.146	-0.735	0.469
Google News	0.280	0.173	1.615	0.145	0.326	0.339	0.360	0.778	-0.226	1.350
Wikipedia edits	-0.567	0.174	-3.260	0.012	1.329	-0.534	0.577	-0.982	-2.094	0.258
Google Trends	0.959	0.208	4.608	0.002	2.654	0.816	0.591	1.621	-0.267	2.182
**Google Trends**										
Confirmed cases	0.590	0.239	2.469	0.036	0.677	0.407	0.426	1.383	-0.578	0.995
PubMed/MEDLINE	0.007	0.223	0.033	0.974	0.000	0.058	0.296	0.025	-0.716	0.635
Google News	0.268	0.263	1.019	0.335	0.115	0.305	0.332	0.808	-0.466	1.117
Wikipedia edits	0.108	0.277	0.390	0.705	0.017	0.224	0.482	0.224	-0.665	1.368
**Wikipedia**										
Confirmed cases	0.375	0.246	1.521	0.159	0.231	0.411	0.279	1.341	-0.006	0.952
PubMed/MEDLINE	-0.044	0.255	-0.174	0.865	0.003	-0.055	0.307	-0.144	-0.681	0.776
Google News	0.489	0.258	1.898	0.087	0.360	0.378	0.325	1.507	-0.286	1.075
**Google News**										
Confirmed cases	0.223	0.280	0.798	0.442	0.058	0.258	0.269	0.829	-0.182	0.953
PubMed/MEDLINE	0.337	0.280	1.206	0.253	0.132	0.224	0.353	0.956	-0.612	0.872
**PubMed/MEDLINE**										
Confirmed cases	0.278	0.277	1.004	0.335	0.084	0.256	0.192	1.451	-0.079	0.659

**Table 5:** Structural equation modeling for confirmed cases of Chikungunya virus (adjusted model).

Latent variable	Value	Standard error	T	Pr > |t|	f^2^	Value Bootstrap	Standard error Bootstrap	Critical ratio (CR)	Lower bound (95%)	Upper bound (95%)
**Twitter**										
Confirmed cases	0.029	0.102	0.280	0.787	0.010	-0.067	1.322	0.022	-0.566	0.968
PubMed/MEDLINE	-0.107	0.098	-1.092	0.307	0.149	0.045	0.516	-0.208	-0.420	0.782
Google News	0.855	0.296	2.894	0.020	1.047	0.485	2.355	0.363	-3.019	2.468
Wikipedia edits	-0.634	0.232	-2.730	0.026	0.932	-0.284	3.892	-0.163	-3.438	2.303
Google Trends	0.806	0.234	3.451	0.009	1.488	0.660	0.868	0.929	-1.353	3.168
**Google Trends**										
Confirmed cases	0.308	0.104	2.972	0.016	0.981	0.152	0.276	1.116	-0.573	0.520
PubMed/MEDLINE	-0.151	0.131	-1.156	0.277	0.149	0.013	0.216	-0.699	-0.447	0.530
Google News	0.740	0.342	2.165	0.059	0.521	0.474	0.714	1.036	-1.842	1.659
Wikipedia edits	0.227	0.322	0.703	0.500	0.055	0.408	0.727	0.312	-0.689	3.313
**Wikipedia**										
Confirmed cases	0.098	0.097	1.008	0.337	0.102	-0.119	0.228	0.429	-0.892	0.375
PubMed/MEDLINE	-0.079	0.126	-0.627	0.545	0.039	0.042	0.198	-0.397	-0.376	0.562
Google News	0.988	0.122	8.103	0.000	6.566	0.938	0.221	4.461	0.261	1.689
**Google News**										
Confirmed cases	-0.044	0.240	-0.182	0.859	0.003	0.001	0.353	-0.124	-0.895	0.626
PubMed/MEDLINE	0.657	0.240	2.741	0.019	0.683	0.495	0.340	1.932	-0.414	0.991
**PubMed/MEDLINE**										
Confirmed cases	0.278	0.277	1.004	0.335	0.084	0.277	0.233	1.193	-0.092	0.792

**Table 6:** Structural equation modeling for notified cases of Chikungunya virus (unadjusted model).

Latent variable	Value	Standard error	T	Pr > |t|	f^2^	Value Bootstrap	Standard error Bootstrap	Critical ratio (CR)	Lower bound (95%)	Upper bound (95%)
**Twitter**										
Notified cases	0.118	0.210	0.561	0.590	0.039	0.081	0.481	0.244	-0.888	1.098
PubMed/MEDLINE	0.038	0.138	0.280	0.787	0.010	-0.043	0.237	0.162	-0.732	0.525
Google News	0.287	0.170	1.693	0.129	0.358	0.341	0.370	0.776	-0.503	1.276
Wikipedia edits	-0.571	0.169	-3.385	0.010	1.432	-0.582	0.532	-1.074	-2.112	0.223
Google Trends	0.907	0.226	4.014	0.004	2.014	0.727	0.579	1.566	-0.389	1.806
**Google Trends**										
Notified cases	0.662	0.217	3.051	0.014	1.034	0.502	0.393	1.684	-0.259	1.109
PubMed/MEDLINE	-0.015	0.203	-0.072	0.944	0.001	0.011	0.237	-0.062	-0.639	0.458
Google News	0.228	0.239	0.954	0.365	0.101	0.242	0.283	0.804	-0.462	0.820
Wikipedia edits	0.107	0.246	0.433	0.675	0.021	0.233	0.413	0.258	-0.565	1.201
**Wikipedia**										
Notified cases	0.348	0.256	1.362	0.203	0.186	0.386	0.307	1.135	-0.075	1.195
PubMed/MEDLINE	-0.044	0.260	-0.171	0.868	0.003	-0.014	0.328	-0.135	-0.633	0.753
Google News	0.483	0.265	1.821	0.099	0.332	0.368	0.346	1.396	-0.308	1.155
**Google News**										
Notified cases	0.260	0.280	0.931	0.372	0.079	0.308	0.369	0.705	-0.231	1.169
PubMed/MEDLINE	0.320	0.280	1.144	0.277	0.119	0.233	0.418	0.764	-0.614	0.888
**PubMed/MEDLINE**										
Notified cases	0.306	0.275	1.115	0.287	0.104	0.322	0.214	1.431	0.000	0.829

**Table 7:** Structural equation modeling for notified cases of Chikungunya virus (adjusted model).

Latent variable	Value	Standard error	t	Pr > |t|	f^2^	Value Bootstrap	Standard error Bootstrap	Critical ratio (CR)	Lower bound (95%)	Upper bound (95%)
**Twitter**										
Notified cases	0.039	0.111	0.348	0.737	0.015	0.058	0.388	0.100	-0.436	0.982
PubMed/MEDLINE	-0.113	0.101	-1.126	0.293	0.159	-0.027	0.347	-0.327	-1.064	0.315
Google News	0.858	0.293	2.926	0.019	1.070	0.285	1.113	0.771	-3.287	3.360
Wikipedia edits	-0.620	0.238	-2.610	0.031	0.851	0.257	1.739	-0.357	-1.741	7.822
Google Trends	0.787	0.255	3.086	0.015	1.191	0.440	1.477	0.533	-3.430	3.445
**Google Trends**										
Notified cases	0.327	0.096	3.395	0.008	1.281	0.195	0.242	1.353	-0.536	0.597
PubMed/MEDLINE	-0.147	0.122	-1.204	0.259	0.161	-0.009	0.170	-0.866	-0.399	0.356
Google News	0.661	0.314	2.109	0.064	0.494	0.429	0.609	1.086	-0.759	1.643
Wikipedia edits	0.279	0.296	0.941	0.371	0.098	0.437	0.609	0.458	-0.749	1.522
**Wikipedia**										
Notified cases	0.077	0.100	0.768	0.460	0.059	-0.116	0.165	0.466	-0.548	0.159
PubMed/MEDLINE	-0.074	0.128	-0.580	0.575	0.034	0.052	0.183	-0.407	-0.287	0.545
Google News	0.982	0.125	7.861	0.000	6.180	0.924	0.193	5.097	0.373	1.266
**Google News**										
Notified cases	0.012	0.241	0.049	0.962	0.000	0.050	0.348	0.034	-0.606	0.621
PubMed/MEDLINE	0.643	0.241	2.665	0.022	0.646	0.514	0.268	2.399	-0.391	0.920
**PubMed/MEDLINE**										
Notified cases	0.306	0.275	1.115	0.287	0.104	0.287	0.205	1.496	-0.054	0.598

**Table 8:** Structural equation modeling for allochthonous/ imported cases of Chikungunya virus (unadjusted model).

Latent variable	Value	Standard error	t	Pr > |t|	f^2^	Value Bootstrap	Standard error Bootstrap	Critical ratio (CR)	Lower bound (95%)	Upper bound (95%)
**Twitter**										
Imported cases	-0.054	0.131	-0.413	0.691	0.021	0.220	0.470	-0.115	-0.232	1.918
PubMed/MEDLINE	0.037	0.141	0.265	0.798	0.009	0.061	0.286	0.131	-0.620	0.662
Google News	0.284	0.172	1.651	0.137	0.341	0.300	0.547	0.518	-1.138	1.136
Wikipedia edits	-0.556	0.168	-3.314	0.011	1.373	-0.497	0.366	-1.518	-1.786	0.128
Google Trends	0.987	0.162	6.097	0.000	4.647	0.562	0.770	1.281	-1.453	1.931
**Google Trends**										
Imported cases	-0.128	0.267	-0.480	0.642	0.026	0.002	0.311	-0.412	-0.728	0.854
PubMed/MEDLINE	0.073	0.289	0.253	0.806	0.007	0.101	0.276	0.265	-0.660	0.669
Google News	0.272	0.342	0.794	0.447	0.070	0.245	0.389	0.699	-0.636	1.044
Wikipedia edits	0.392	0.320	1.225	0.252	0.167	0.485	0.338	1.159	-0.269	1.240
**Wikipedia**										
Imported cases	-0.067	0.263	-0.254	0.805	0.006	-0.085	0.158	-0.423	-0.485	0.273
PubMed/MEDLINE	0.005	0.286	0.019	0.985	0.000	0.033	0.335	0.016	-0.729	0.745
Google News	0.594	0.282	2.107	0.061	0.444	0.517	0.248	2.394	-0.232	0.895
**Google News**										
Imported cases	0.164	0.277	0.593	0.565	0.032	0.191	0.246	0.669	-0.498	0.702
PubMed/MEDLINE	0.431	0.277	1.554	0.148	0.220	0.288	0.368	1.169	-0.601	0.862
**PubMed/MEDLINE**										
Imported cases	-0.190	0.283	-0.671	0.515	0.038	-0.220	0.193	-0.988	-0.711	0.115

**Table 9:** Structural equation modeling for allochthonous/ imported cases of Chikungunya virus (adjusted model).

Latent variable	Value	Standard error	t	Pr > |t|	f^2^	Value Bootstrap	Standard error Bootstrap	Critical ratio (CR)	Lower bound (95%)	Upper bound (95%)
**Twitter**										
Imported cases	-0.072	0.045	-1.610	0.146	0.324	0.025	0.247	-0.292	-0.576	0.522
PubMed/MEDLINE	-0.105	0.056	-1.882	0.097	0.443	-0.020	0.157	-0.668	-0.381	0.362
Google News	1.565	0.451	3.472	0.008	1.507	0.354	1.343	1.165	-3.680	3.565
Wikipedia edits	-1.412	0.421	-3.354	0.010	1.406	0.015	1.299	-1.087	-2.922	2.926
Google Trends	0.894	0.173	5.179	0.001	3.353	0.600	1.209	0.740	-1.868	3.274
**Google Trends**										
Imported cases	-0.056	0.084	-0.662	0.525	0.049	-0.059	0.145	-0.384	-0.410	0.280
PubMed/MEDLINE	-0.013	0.108	-0.120	0.907	0.002	-0.011	0.220	-0.059	-0.548	0.446
Google News	0.721	0.836	0.863	0.411	0.083	0.556	0.577	1.251	-0.529	2.254
Wikipedia edits	0.263	0.808	0.326	0.752	0.012	0.449	0.543	0.485	-1.206	1.465
**Wikipedia**										
Imported cases	-0.026	0.032	-0.814	0.434	0.066	0.038	0.145	-0.179	-0.275	0.398
PubMed/MEDLINE	-0.053	0.039	-1.381	0.197	0.191	0.065	0.185	-0.289	-0.348	0.475
Google News	1.027	0.038	26.949	0.000	72.625	0.933	0.222	4.626	0.602	1.352
**Google News**										
Imported cases	0.216	0.244	0.887	0.394	0.071	0.092	0.337	0.641	-0.803	0.693
PubMed/MEDLINE	0.611	0.244	2.505	0.029	0.570	0.486	0.374	1.633	-0.439	1.101
**PubMed/MEDLINE**										
Imported cases	-0.190	0.283	-0.671	0.515	0.038	-0.253	0.223	-0.852	-0.694	0.132

## Discussion

The surveillance of disease outbreaks and their correlation to web searches was addressed in multiple occasions in the medical literature [26, 27]. Recently, an outbreak of Chikungunya was recorded in the Lazio region (western central part of Italy). This ongoing outbreak, which started in August 2017, has provoked public awareness as reflected by high peaks of web-related activity as shown here in our study.

Similarly, a burst of web related searches was recorded by GT mirroring the previous Chikungunya outbreak in Italy ten years ago; a modest peak of GT searches corresponded to the reported cases of Chikungunya in 2014 from the Caribbean and Central America [28]. Interestingly, the current outbreak is met with a greater public interest. The rise in cases is accompanied with an accelerated upslope of Google-related searches that are increasing faster than that seen in 2007 (which remain significant after adjusting for the growth of Internet users throughout the years in the study period).

Chikungunya-related reports in GN revealed news peaks in 2007 and 2017, corresponding to the Chikungunya outbreaks in Italy. Several smaller peaks were recorded during the allochthonous cases mentioned earlier, supporting the fact that local cases induce a more significant impact on web search activity. The role of GN during outbreaks was recently shown concerning the Zika virus outbreak reporting an increase in GN-related Zika outbreak web searches underlying worries and concerns of the public [29].

Infoveillance can be appreciated from other web-based search engines too. Wikipedia is a prominent online health source of information that has been shown to have an integral role in increasing public knowledge concerning the emergence of new disease or outbreaks of infectious agents [30, 31]. Currently, the Italian public interest has been clearly reflected by the large volume of Wikipedia page visits during the present Chikungunya outbreak. Similar results were reported following the 2015 outbreak of Zika virus in Central and South America [29]. Moreover, high levels of public health concerns were documented by increased Wikipedia page visits around the announcement of H1N1 vaccine outbreak back in 2012 [32]. Furthermore, the high number of edits in the Italian “Chikungunya” Wikipedia page reported herein also reflect the significant interest of volunteer editors in the matter.

Twitter is another fundamental social media data stream that is a highly used by the public to share information, including health related issues. In our study, the increase of public awareness was demonstrated by increased Twitter activity. Being launched only in July 2006, it is reasonable that the 2007 Chikungunya outbreak in Italy won no attention in comparison with the large spike of activity of the current outbreak. This can be attributed to the very few users (around 702,000) on the platform during its first years since inception, which was followed by an exponential growth in users in the beginning of 2009 reaching over 300 million monthly active users in 2017 [33, 34]. Within the past decade, a small spike in Chikungunya-related tweets is documented in 2014, coinciding with the small outbreak of allochthonous cases of Chikungunya [28].

From a scientific standpoint, PubMed/MEDLINE is one of the leading resources for published medical papers. As for scholarly articles concerning Chikungunya published since 2004, the first spike was noted approximately one year after the 2007 Chikungunya outbreak and dropped immediately afterward. The delay of one year between the outbreak and publication peak might be attributed to the time frame it takes for manuscripts to be peer-reviewed, processed and published. Similarly, major articles describing the Ebola outbreak in 2014 came to light half a year following the outbreak official announcement by the WHO in March of the same year [35, 36].

Subsequently, smaller peaks of published research were recorded between the 2007 and 2017 outbreaks, coinciding with Chikungunya infection in returned travelers to Italy. Prior to the current outbreak, the largest number of allochthonous cases of Chikungunya in Italy were recorded during the years 2014–2015, thus contributing to the peak of publications in 2016 [28]. In June 2016, Guzetta et al. [37] discussed the potential risk of Chikungunya and dengue outbreaks in northern Italy using a mathematical model which was built based on mosquito abundance data. The authors estimated the potential of imported human cases of Chikungunya or dengue to generate autochthonous cases in Italy in the absence of control interventions. The current outbreak fits with the findings of the latter study in terms of timing. Further investigations at the end of the current outbreak would provide better data facilitating the comparison between the 2007 and 2017 outbreak in terms of publications in PubMed/MEDLINE, taking into consideration the unavoidable delay between outbreaks and publishing.

Addressing the interaction between the novel data streams using structural equation modelling, we found that cases, whether notified or confirmed, positively affected GT in terms of search volume. In addition, we found them both to positively affect Twitter in terms of tweets. To better elucidate the last points, users tended to search for “Chikungunya” as a topic on Google before posting about it on Twitter. On the other hand, editing Wikipedia pages was found to negatively affect (suppress) the volume of tweets. Imported cases of Chikungunya had no effect on GT. Nevertheless, the effect of Wikipedia editing on Twitter posts was found to be negative.

Another interesting finding we found using the adjusted models was that for all Chikungunya case models, edits to Wikipedia pages and posts on Twitter were positively affected by PubMed/MEDLINE publications, with GN articles acting as a mediator. In other words, as more articles were published in PubMed/MEDLINE concerning Chikungunya, more articles were released into public news streams as shown by GN, and in result, more tweets were posted and the Chikungunya-related Wikipedia page was edited to reflect these updated and news. To the best of our knowledge, no similar studies have addressed this issue/topic using structural equations with such interplay of novel data streams. In the extant literature, only Rodgers et al. [38] have assessed the usage of media sources as variables for the statistical analysis to predict public health behaviors showing that there is predictive value for including media variables as part of the segmentation process. Another study also noted similar increases in page views and edits on Wikipedia articles that were related to certain topics featured on TV or news outlets [39].

The importance of these findings would better guide health authorities to take advantage of web streams in terms of providing credited news as well as addressing the population’s concerns during outbreaks. The use of online content to detect public interest and disease outbreaks have been shown to be quicker than traditional public health surveillance, thus providing prospects for revolutionizing future surveillance methodologies [40].

Our study has several limitations; first, the precise algorithms used by GT, GN, Wikipedia, Twitter and PubMed/MEDLINE are not publicly available, leading to difficulties in processing and manipulation of the data, as already reported. Additionally, the presented data are provided as relative, normalized figures, and not as absolute, crude data. Therefore, our results and findings strictly depend on the chosen time window and geographic location studied. However, we compared GT, GN, Wikipedia, Twitter, and PubMed/MEDLINE search volumes to each other, showing that the behavior is consistent and reproducible among different web platforms. Furthermore, it remained significant also after adjusting for the Internet penetration index.

In conclusion, exploiting novel online data streams provide public health professionals the tools to detect disease outbreak patterns earlier, and thus to employ more effective policies and measures.

However, despite great progresses made regarding the internet as a major source of health-related information, there are still many challenging issues frequently encountered, including the future implications of such novel data streams as an effective tool to prevent infectious diseases outbreaks. Therefore, nationwide public health authorities, particularly Italian health authorities, should take advantage of our findings in dealing with the current outbreak of Chikungunya in Italy. Moreover, health authorities should be aware of the public’s reactions to current events, to recognize online resources as tools for collecting the concerns of public opinion and reply to them, disseminating awareness and avoid misleading information.
