# Correction: Public reaction to Chikungunya outbreaks in Italy—Insights from an extensive novel data streams-based structural equation modeling analysis

**DOI:** 10.1371/journal.pone.0222865

**Published:** 2019-09-17

**Authors:** Naim Mahroum, Mohammad Adawi, Kassem Sharif, Roy Waknin, Hussein Mahagna, Bishara Bisharat, Mahmud Mahamid, Arsalan Abu-Much, Howard Amital, Nicola Luigi Bragazzi, Abdulla Watad

The tenth author's initials are indexed incorrectly in PubMed. The correct initials are: Bragazzi NL.
